# Trimetazidine stimulates intracellular Ca^2+^ transients and zebrafish locomotor activity in spinal neurons

**DOI:** 10.1038/s41598-025-06065-y

**Published:** 2025-07-02

**Authors:** Sara Bernardi, Sara Vitolo, Chiara Gabellini, Maria Marchese, Elisabetta Ferraro

**Affiliations:** 1 Neurobiology and Molecular Medicine Unit, IRCCS Fondazione Stella Maris, Via dei Giacinti, 2, Calambrone, Pisa, 56128 Italy; 2 Department of Biology, Unit of Cellular, Molecular and Developmental Biology, University of Pisa, S.S. 12 Abetone e Brennero 4, Pisa, 56127 Italy

**Keywords:** Amyotrophic lateral sclerosis, Trimetazidine, Aging, Spinal cord, Skeletal muscle, Motor neuron disease, Neuro-muscular junction, Amyotrophic lateral sclerosis, Neuromuscular junction, Spinal cord, Ageing, Target identification

## Abstract

The metabolic modulator trimetazidine (TMZ) is an antianginal recently found to improve skeletal muscle performance in mice models of sarcopenia and of amyotrophic lateral sclerosis (ALS). The mechanism underlying the effect of TMZ on locomotor activity has been proposed to rely on its ability to enhance metabolic efficiency with a consequent improvement of myogenesis and of neuromuscular junction (NMJ) and muscle function. However, although promising and therefore under clinical trials, the mechanism of action of TMZ has not been clearly disclosed; here we hypothesized that it might involve the modulation of neuronal Ca^2+^ flows. We studied the effect of TMZ on Ca^2+^ dynamics in vivo, by using the transgenic zebrafish line *Tg(neurod1*:GCaMP6f*)* in which the neuronal expression of the Ca^2+^ indicator GCaMP allows to visualize Ca^2+^ dynamics in neurons of zebrafish larvae. By this elegant tool, we demonstrated, for the first time, that TMZ promotes an increase of intracellular Ca^2+^ transients in zebrafish spinal neurons likely enhancing motor neuron firing, which correlates with enhanced motor performance induced by this drug. Even though elevated intracellular Ca^2+^ levels have often been associated to neurotoxicity, it is unclear if the neuronal excitability features in some neuro-muscular disorders are compensatory or pathological. Therefore, this newly reported effect of TMZ which transiently and selectively enhances spinal neuron firing deserves to be further detailed and taken into account when the possible repurposing of this drug is proposed for the treatment of neuro-muscular disorders.

**Supplementary Information:**

The online version contains supplementary material available at 10.1038/s41598-025-06065-y.

## Introduction

Trimetazidine (TMZ) is a metabolic modulator which has been originally developed for the treatment of ischemic heart conditions and is used as an antianginal due to its ability to optimize heart metabolism and to be cytoprotective^1^. Also, it has more recently been demonstrated that TMZ acts on skeletal muscle by promoting myogenesis, by improving muscle quality and neuromuscular communication, and by increasing muscle strength in mice models of aging and cachexia, as well as in patients with peripheral arterial disease^2–5^. Moreover, we have reported that orally administered TMZ leads to motor performance improvement in transgenic SOD1^G93A^ mice, the standard animal model for amyotrophic lateral sclerosis (ALS), a neurodegenerative disease affecting motor neurons^6^.

Given that mitochondrial and metabolic dysfunctions are among the pathogenic mechanisms proposed to explain the origin of ALS^7–9^we have recently focused our attention on assessing the preclinical efficacy of the metabolic modulator TMZ in counteracting ALS progression^6^. TMZ is a piperazine derivative partially inhibiting the 3-ketoacyl coenzyme A thiolase, an essential enzyme involved in long-chain fatty acid β-oxidation. This leads to a shift from fatty acid to glucose as preferentially used substrate, thus glycolysis to glucose oxidation coupling is improved. This metabolic shift has been found to optimize cell energy production, especially under conditions of limited oxygen availability. In fact, ATP synthesis through fatty acid β-oxidation requires more oxygen compared with glucose oxidation; therefore, the choice of glucose as a substrate induces a more efficient utilization of the oxygen available, which in turn increases metabolic efficiency^10–12^. However, evidence supporting this mechanism remains somewhat controversial, and since, based on its preclinical efficacy, TMZ is under clinical trial (NCT04788745), elucidating the details of its mode of action is of high relevance. Moreover, we had previously observed that murine skeletal muscles treated with TMZ ex vivo display a very quick shift towards a slow-twitch contractile phenotype -typically relying on a mitochondrial oxidative metabolism rather than on glycolysis^3^. Even though this might sound counterintuitive -being TMZ an agent reducing β-oxidation - this very rapid effect might not be explained by gene expression modulation therefore we hypothesized an ability of this drug to modify ionic fluxes. To address this issue, we focused our attention on Ca^2+^ since it is the final ionic effector of both skeletal muscle contraction and motor neuron firing. This choice was also supported by previous studies reporting that TMZ affects Ca^2+^ handling in cardiomyocytes, although the precise molecular mechanisms remain unclear^13–16^.

In order to elucidate the beneficial effect of TMZ on motor performance, we chose to study the role of this drug on Ca^2+^ dynamics in neurons and neuronal activity in vivo, and we took advantage of a model represented by the transgenic zebrafish line *Tg(neurod1*:GCaMP6f*)* for in vivo Ca^2+^ imaging in neurons^17^. By this tool we visualized Ca^2+^ dynamics in neuronal cells demonstrating the ability of TMZ to induce intracellular Ca^2+^ transients and spinal neuron firing which correlates with enhanced locomotion in TMZ-treated zebrafish larvae.

## Results

### TMZ stimulates locomotor activity in zebrafish larvae

**Fig. 1:**
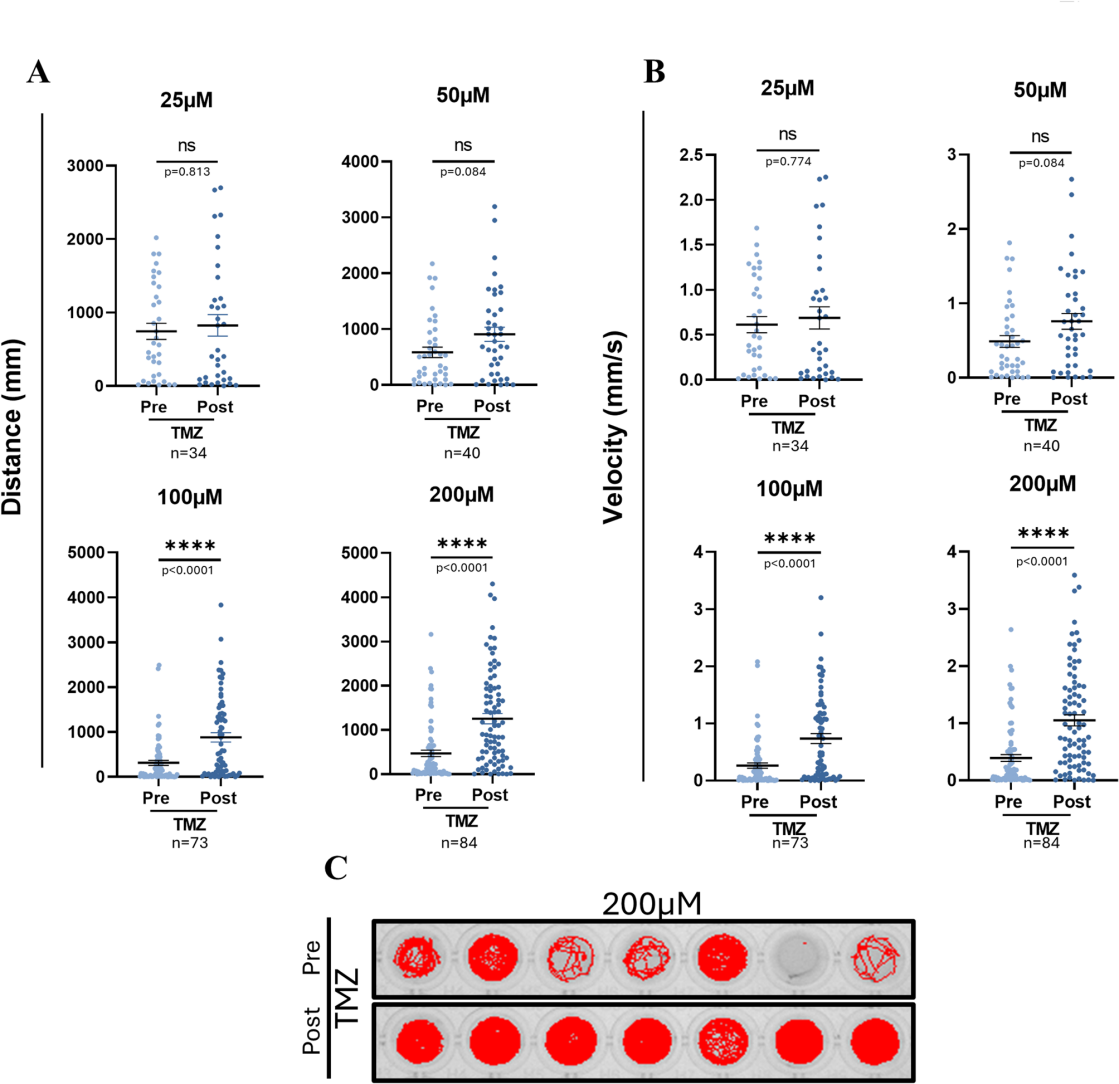
Locomotor behavior in TMZ-treated zebrafish larvae. (**A**) Distance traveled (mm) and (**B**) velocity (mm/s) of zebrafish larvae pre- and post-TMZ administration at increasing TMZ concentrations. (**C**) Representative movement track pre- and post-TMZ (200µM) administration. n range = 34–84 larvae/group; specifically indicated in each panel. Statistical analysis was performed using the paired Wilcoxon test- Asterisks denote significance; ****p ≤ 0.0001). Data are presented as Mean ± SEM.

In order to confirm the effect of TMZ on locomotion also on zebrafish larvae, which we used as model to evaluate Ca^2+^ fluxes, we first performed a toxicity assay using increasing concentrations of TMZ, specifically 25 µM, 50 µM, 100 µM, 200 µM, 1000 µM and 2000 µM. We administered the drug to 4 dpf (days post fertilization) larvae and we kept them in these solutions for 24 h. We observed that 1000 µM and 2000 µM TMZ were toxic for the larvae which died a few hours upon drug administration while lower dosages did not affect the survival (data not shown). For this reason, we performed the behavior locomotor assay by administrating 25 µM, 50 µM, 100 µM and 200 µM TMZ to 4 dpf larvae to obtain the velocity and the distance travelled by the larvae pre- and post-TMZ treatment (Fig. 1). Our data confirm that there is a statistically significant increase of the motor function upon both 100 µM and 200 µM TMZ treatment (Fig. 1A, B). These data are in line with the results obtained by treating the Tg SOD1^G93A^ mouse model of ALS and that of aging with TMZ which improved their locomotory activity^2,6^ and make zebrafish larvae a suitable model to unravel the mechanisms underlying TMZ effect on locomotion.

### Intracellular Ca^2+^ transients in spinal cord are stimulated by TMZ

Based on the analysis of zebrafish locomotor behaviour upon TMZ treatment, along with the previous observation that ex vivo administration of 100 µM TMZ is able to act very quickly on the contraction rate of excised *tibialis anterior* murine muscles^3^we hypothesized that this metabolic modulator might be able to act on skeletal muscle contractility and, consequently, on locomotion, by modifying ionic fluxes. Therefore, we decided to evaluate the firing of the skeletal muscle-stimulating motor neurons through the analysis of Ca^2+^ fluxes in vivo. We used the transgenic zebrafish line *Tg(neurod1*:GCaMP6f*)* based on the *neurod1* promoter-mediated pan-neuronal expression of the GFP-based Ca^2+^ indicator GCaMP6f.

**Fig. 2:**
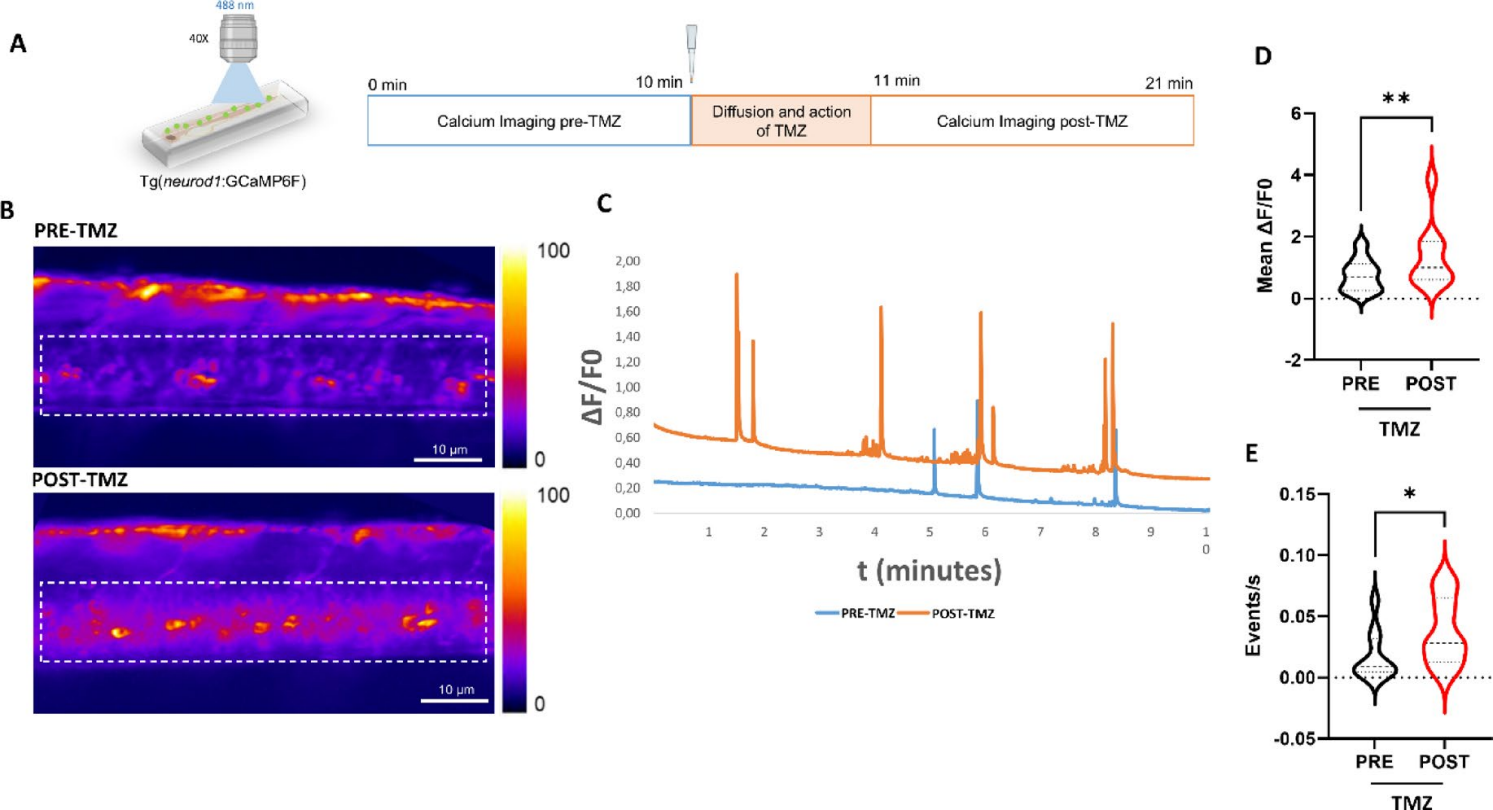
Spinal cord Ca^2+^ imaging upon TMZ exposure. (**A**) Ca^2+^ imaging in spinal cord neurons of Tg(*neurod1*:GCaMP6f) larvae was performed 10 minutes (min) before 100 µM TMZ treatment for baseline recordings (PRE-TMZ). After TMZ administration and after a TMZ-diffusion time of 1 min, calcium imaging was resumed for other 10 min (POST-TMZ). (**B**) Representative z-projection images of the spinal cord Ca^2+^ imaging performed in the lateral caudal neurons of a single zebrafish larva before the treatment (PRE-TMZ) and after the treatment (POST-TMZ), the Region of Interest (ROI) is highlighted with the white dotted line). On the right side, the look up table color range of Ca^2+^ fluorescence is shown. (**C**) A representative graph from a single zebrafish larva shows the ∆F/F0 calcium fluorescent signal in spinal cord for each frame before and after TMZ treatment (**D**) The graph shows the full distribution of data with the median and quartiles of ∆F/F0 fluorescent signal in spinal neurons. (**E**) The graph shows full distribution of data, with median and quartiles of frequency fluorescent signal in spinal neurons *n* = 10 analyzed larvae. Statistical analysis was performed using the paired Wilcoxon test. Asterisks denote significance; *p ≤ 0.05, **p ≤ 0.01 (p of ∆F/F0 = 0.003; p of frequency = 0.01).

100 µM TMZ was administered to 4 dpf Tg(*neurod1*:GCaMP6f) larvae and Ca^2+^ imaging was first performed on spinal cord (Figs. 2 and 40X magnification). The images were acquired before and after TMZ treatment with the timing described in Fig. 2A. By comparing the fluorescence signal derived from GCaMP activation before and after TMZ stimulation, a significant increase in intracellular Ca^2+^ -and thus neuron firing- was observed upon TMZ administration (see the representative images in Fig. 2B and video recordings in Supplementary Video S1). Accordingly, the ∆F/F0 ratio over time shows a higher fluorescent signal recorded following TMZ treatment (Fig. 2C; POST-TMZ) compared to the signal obtained from the untreated larvae (Fig. 2C; PRE-TMZ). The statistical analyses confirms that TMZ was effective in increasing intracellular Ca^2+^ transients in neurons of the spinal cord where motor neurons are localized (Fig. 2D). Ca^2+^ influx was further analysed by assessing the frequency (events/seconds) and duration (seconds) of individual events. Statistical analyses show that post-treatment larvae also exhibited a significant increase in event frequency, while event duration remained unchanged (Fig. 2E and Supplementary Fig. 1A). Also spinal interneurons modulating motor neuron activity are highlighted in this Tg model, therefore, by this experiment it is not possible to precisely identify the neuronal population targeted by TMZ which might likely be represented by motor neurons (indeed is it possible to observe the axon leaving the spinal cord, which indicates that motor neurons are involved; see Fig. 2B and Supplementary video S1) or by interneurons modulating motor neuron activity.

### TMZ does not induce an overall brain increase of intracellular Ca^2+^ transients

**Fig. 3:**
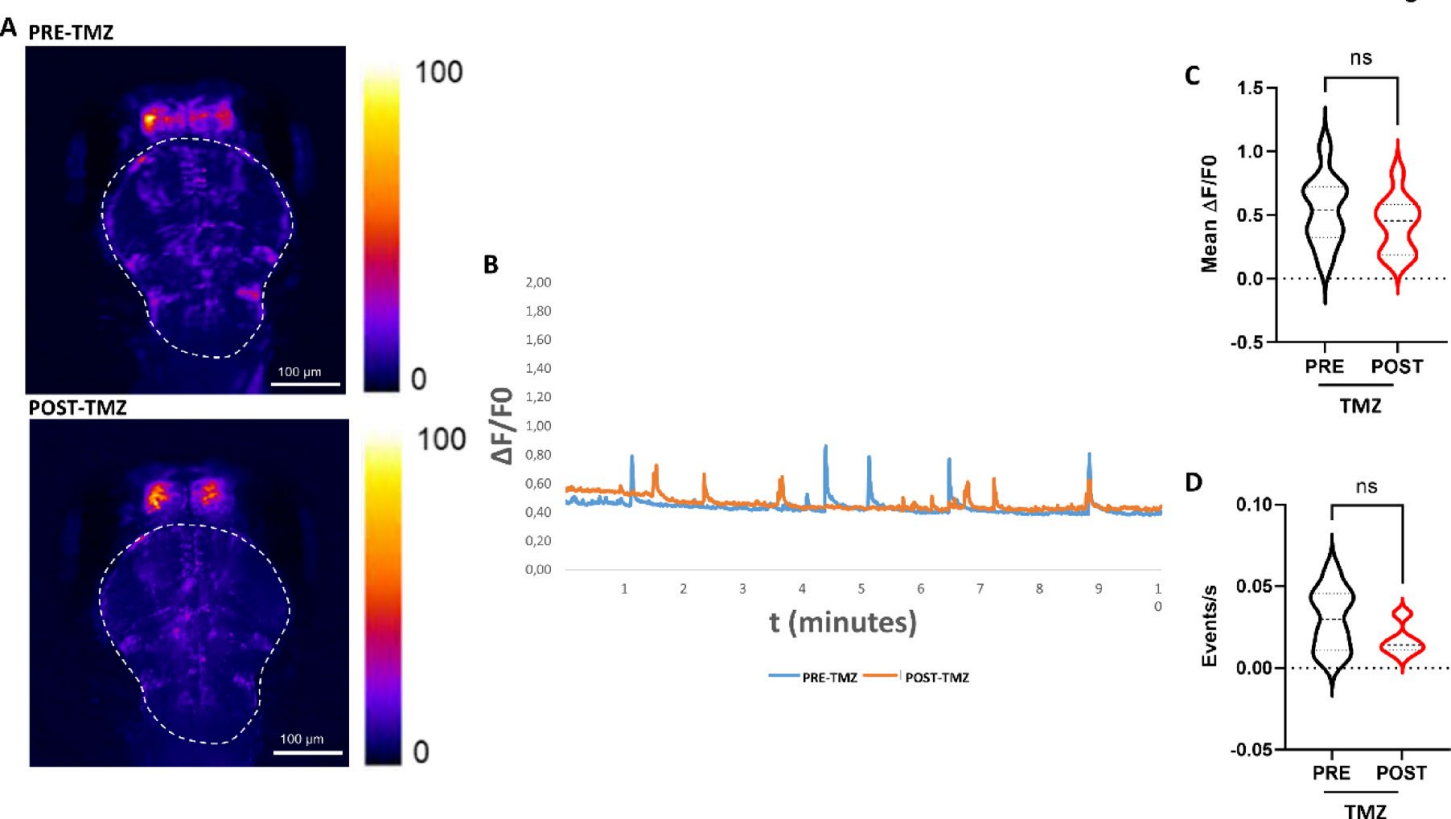
Whole brain Ca^2+^ imaging upon TMZ exposure. (**A**) Representative z-projection images of the whole brain Ca^2+^ imaging performed in the rostral-dorsally placed Tg(*neurod1*:GCaMP6f) larvae before the treatment (PRE-TMZ) and after the treatment (POST-TMZ). On the right side, the look up table color range of Ca^2+^ fluorescence is shown. Images were taken with 4X magnification objective. The dotted line indicates the analyzed ROI. (**B**) A representative graph from a single zebrafish larva shows the ∆F/F0 Ca^2+^ fluorescent signal in the whole brain for each frame before and after TMZ treatment (**C**) The graph shows full distribution of data, with median and quartiles of ∆F/F0 fluorescent signal in whole brain. (**D**) The graph shows full distribution of data, with median and quartiles of frequency fluorescent signal in whole brain. *n* = 10 analyzed larvae. Since the same individuals were analyzed before and after the treatment, statistical analysis was performed using the paired Wilcoxon test. ns: not significant, (p of ∆F/F0 = 0.19; p of frequency = 0.06;).

In order to evaluate if the effect of TMZ was specific to the spinal cord or was occurring in all CNS neurons, we visualized whole brain activity by recording Ca^2+^ imaging at lower magnification (4X). The images were acquired before and after TMZ treatment with the timing described in Fig. 2A. Differently from what we had previously observed in the spinal cord (Fig. 2), no increase in the whole brain intracellular Ca^2+^ transients was observed after TMZ treatment; see representative images (Fig. 3A,B), videos (Supplementary video S2) and graphs (Fig. 3C,D; Supplementary Fig. 1B). The analysis of Ca^2+^ influx in the olfactory bulb was performed separately from the rest of the brain, as this region exhibits a much higher baseline fluorescence and remains continuously active (Supplementary Figure S2), potentially masking changes occurring in other brain areas. Also in the olfactory bulb, both the trace and the graphs clearly show the lack of variation following TMZ treatment (Supplementary Figure S2B,C).

**Fig. 4:**
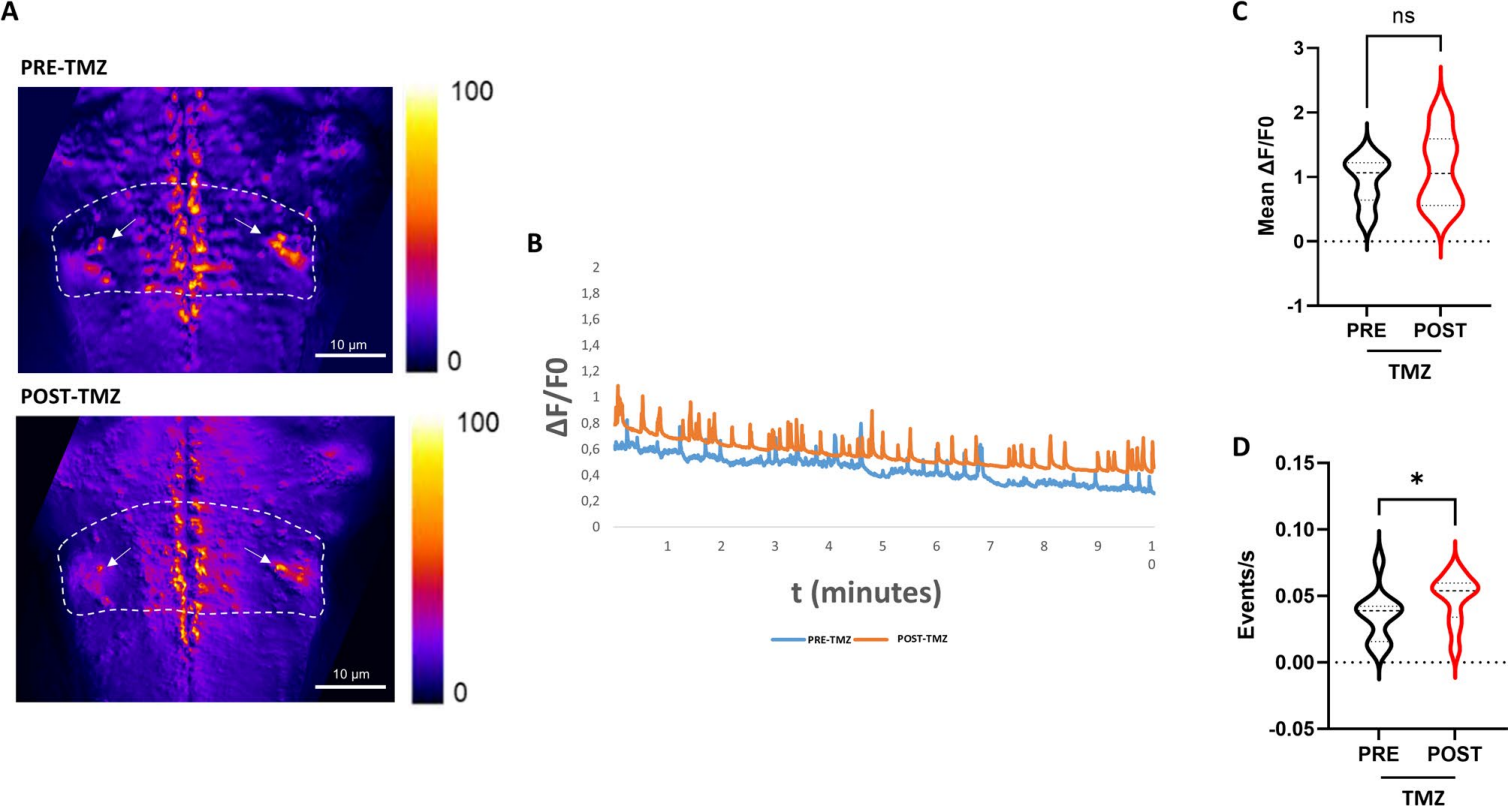
Hindbrain Ca^2+^ imaging upon TMZ exposure. (**A**) Representative z-projection images of the hindbrain calcium imaging performed in Tg(*neurod1*:GCaMP6f) larvae rostral-dorsally settled before the treatment (PRE-TMZ) and after the treatment (POST-TMZ). On the right side, the look up table color range of Ca^2+^ fluorescence is shown. The dotted line indicates the analyzed ROI, while the white arrows indicate the Mauthner cells localization. (**B**) A representative graph from a single zebrafish larva shows the ∆F/F0 Ca^2+^ fluorescent signal in hindbrain for each frame before and after TMZ treatment. (**C**) The graph shows full distribution of data, with median and quartiles of ∆F/F0 fluorescent signal in in the hindbrain. (**D**) The graph shows full distribution of data, with median and quartiles of frequency fluorescent signal in in the hindbrain *n* = 10 analyzed larvae. Statistical analysis was performed using the paired Wilcoxon test. *p ≤ 0.05, ns: not significant (p of ∆F/F0 = 0.13; p of frequency = 0.03).

However, we decided to focus more specifically on the hindbrain region where neurons contacting and modulating motor neurons are localized, in order to get information on the kind of neurons targeted by TMZ which might be directly targeted motor neurons or spinal interneurons or also higher center neurons controlling locomotor movements. For this reason, the same magnification used for spinal cord analysis (40X) was used for the hindbrain recordings. The region chosen for the analysis is an area including the Mauthner cells within the hindbrain (defined by a dotted line in Fig. 4A). This analysis revealed no statistically significant changes in intracellular Ca^2+^ event amplitude ΔF/F0 and duration in the hindbrain region of Tg(*neurod1*:GCaMP6f) larvae upon TMZ administration (see the Supplementary video S3, the trace and the representative ΔF/F0 graph in Fig. 4B C and Supplementary Fig. 1C). However, the frequency of events in the hindbrain region is significantly higher post-TMZ (see the graph in Fig. 4D). Considering that the Mauthner cells region is involved in fish escape response, this suggests that TMZ might also act on hindbrain neurons involved in the upstream motor circuitry controlling motor neurons.

## Discussion

This study presents, to our knowledge, the first compelling evidence that TMZ significantly enhances Ca^2+^ transients in spinal neurons, indicating a potential role for this drug in modulating Ca^2+^ dynamics. These data also reveal an interesting specificity of TMZ effect, which selectively affects spinal neurons without increasing the overall neuronal excitability, thus suggesting a particular effectiveness in modulating motor control. In motor neuron diseases, where the specific motor neuronal population is affected, such targeted action could be advantageous in preserving motor function without exacerbating excitotoxicity or unwanted effect related to a global increase of neuronal activity. Although the GCaMP6f pan-neuronal expression driven by the *neurod1* promoter does not allow to distinguish motor neuron somata from large spinal interneurons modulating motor neuron activity, the observation of the axon leaving the spinal cord (Fig. 2B; Supplementary video S1) indicate that motor neurons are involved. However, further investigation is needed to confirm the specific spinal neuronal population targeted by TMZ; to elucidate this issue, transgenic zebrafish lines for in vivo Ca^2+^ imaging in specific spinal neuron populations would be helpful.

The increase of Ca^2+^ transients induced by TMZ in spinal neurons may contribute to explaining the enhanced locomotion parameters induced by TMZ on aging and ALS mouse models^2,6^ as also observed in this study on zebrafish larvae. Although this specific effect of TMZ on Ca^2+^ needs to be confirmed in animal models of aging and ALS (such as stable transgenic zebrafish lines recapitulating key features of human C9orf72 and SOD1^G93A^ ALS), the fact that, based on its preclinical efficacy, TMZ has grabbed the attention of the scientific community for the treatment of ALS (clinical trial NCT04788745) deserves a thorough examination of the possible implications of TMZ effect on Ca^2+^ transients, in order to better evaluate its reappraisal as a specific treatment to slow down the progression of motor symptoms in this disease. In the context of ALS, where motor neuron degeneration and progressive loss of muscle function occur^18–23^, TMZ’s ability to improve locomotion is noteworthy. However, it has been reported that elevated intracellular Ca^2+^ levels contribute to neurotoxicity, and that altered Ca^2+^ homeostasis is a pathological feature of ALS^24,25^. On the other hand, even though hyperexcitability and fasciculations are features of ALS^26–30^ -and therefore increased excitability triggered by TMZ might, at first glance, be considered detrimental- some issues need to be considered.

First of all, hyperexcitability origin and contribution to the pathophysiology of ALS are unknown. Motor neuron electrical signaling initially increases during the early stages of ALS and then it gradually decreases, as neurons degenerate and lose their ability to properly respond to stimuli^17,31–33^. It is known that disassembly and denervation of the neuromuscular junction (NMJ) occur in ALS before motor neuron degeneration, with episodes of reinnervation interpreted as attempts to regenerate NMJ^34^. It has been suggested that, with the progressive dysfunction of lower motor neurons, fasciculations arise in reinnervated motor units while later on, as motor neurons degenerate, fasciculations become less prominent^30^. Hyperexcitability might therefore occur along with compensatory -but insufficient- reinnervation, thus it might not be the cause of neurodegeneration. In this scenario, it can be hypothesized that drugs triggering intracellular Ca^2+^ transients, spinal neuron excitability and consequent skeletal muscle contraction might be compensatory and aiming at counteracting neurodegeneration and avoiding NMJ denervation. This is in accordance with our previous finding that TMZ protects against NMJ impairment^6^.

Another important issue worth to be discussed concerns the occurrence of hypoexcitability in spinal motoneurons of ALS mouse models^35–40^whereas cortical hyperexcitability has often been described as an early feature in ALS possibly contributing to neuronal stress and subsequent degeneration^41–45^. Moreover, it has been reported that motor neurons derived from ALS patients induced pluripotent stem cells exhibit hyperexcitability and/or hypoexcitability^26,33,46–50^and that motor neurons recorded from ALS patients are hypoexcitable^51^. Based on some author’s hypothesis, motor neurons do not develop hyperexcitability before NMJ denervation, but some motor neurons fails to produce sustained firing -and hypoexcitability occurs- despite the fact that their NMJs are still functional^35,37^; hypoexcitability might lead to peripheral disconnection and to reduced functionality of fast fatigable NMJ and hyperexcitability might arise in the remaining resistant neurons thus compensating for the hypoexcitable vulnerable ones^52^. In this view, hypoexcitability might be the earliest pathological sign of some motor neurons -possibly due to the accumulation of misfolded SOD1 proteins- leading to dysfunctional motor units, whereas hyperexcitability might be neuroprotective and compensatory^35–37,53^. To sum up, it is still obscure whether hyperexcitability and hypoexcitability are compensatory or pathological events in ASL^36,38^. Therefore, we believe that the potential beneficial therapeutic action of TMZ, a drug transiently enhancing motor neuron excitability, protecting against the dismantlement of NMJs and improving locomotory abilities^6^ deserves consideration and a thorough investigation.

Finally, this study reveals an increased frequency of intracellular Ca^2+^ transients in the hindbrain, following TMZ treatment. The hindbrain plays a key role in locomotor control which is mediated by reticulospinal networks including Mauthner neurons projecting to spinal motor neurons^54,55^. Thus, these data suggest a potential effect of TMZ on motor circuits upstream of spinal cord. Further investigations will be needed to clarify which neuronal populations are targeted by TMZ and by which mechanisms this drug influences Ca^2+^ signaling, for instance through modulation of specific Ca^2+^ channels or buffering systems. Moreover, the observed regional differences in Ca^2+^ regulation might also depend on a different drug delivery efficiency across various CNS regions. Future work including TMZ uptake assays and molecular profiling will help clarify whether the observed regional effects are due to pharmacokinetic factors or depend on a real cell- specific regulation by TMZ.

In conclusion, by demonstrating the increase of Ca^2+^ transients in spinal motor neurons induced by TMZ, this study suggests a new potential mechanism of action of this drug. Based on its preclinical efficacy in improving motor symptoms in neuro-muscular disorders, TMZ has recently been clinically evaluated for the treatment of ALS, and our findings reveal new aspects of TMZ mechanism of action which needs to be taken into account when considering its clinical reappraisal.

## Materials and methods

### Zebrafish maintenance

Experiments were carried out on a wild-type (WT) AB line and the transgenic [Tg(*neurod1:GCaMP6f*)] line (generously provided by Claire Wyart from the Institut du Cerveau et de la Moelle Épinière, Paris, France)^56^ in the nacre (*mitfa*^*−/−*^) background. We had previously confirmed that there were no behavioral differences between the Tg(*neurod1:GCaMP6f*) line and the standard AB line^57^. Adult zebrafish were kept in tanks with a density of no more than five fish per liter, at a constant temperature of 28 °C, under a 14-hour light/10-hour dark cycle. Fertilized eggs and embryos were cultured at 28.5 °C in egg water prepared with “Instant Ocean” sea salts (60 µg/mL) (Aquarium Systems, Sarrebourg, France) and in E3 medium (292.2 mg NaCl, 12.6 mg KCl, 48.6 mg CaCl_2_, and 39.8 mg MgSO_4_ per 1 L of deionized water), following standard procedures^60^. The fish were staged by hours post fertilization (hpf) or days post fertilization (dpf).

### Locomotor behavior

Larval locomotor behavior (distance traveled and velocity) of WT AB larvae at 4 dpf was assessed using the DanioVision system (Noldus Information Technology). Briefly, individual larvae were placed into 96-well plates with 300 µL of E3 medium per well. The plates were then inserted into the DanioVision system, where larval locomotor activity was recorded for 20 min. After this, TMZ, dissolved in E3 medium, was added to each well at increasing concentrations (25, 50, 100 and 200 µM), and larval locomotor activity was recorded again for 20 min. The recorded data were analyzed using EthoVision XT video-tracking software (Noldus Information Technology) to measure the distance traveled and the velocity of the larvae.

### Ca^2+^ imaging recordings

Zebrafish larvae at 4 dpf were immobilized in low melting point agarose, with the larvae arranged caudal-laterally for spinal cord recordings and rostral-dorsally for whole brain and hindbrain recordings. A Nikon FN1 microscope (Nikon, Tokyo, Japan) was used for video recording, with image acquisitions made using a Prime sCMOS camera (Teledyne Photometrics, Tucson, AZ, USA) and Metafluor software (Molecular Devices, San Jose, CA). A time-lapse interval of 150 ms was applied, capturing 2412 frames per video. The recordings were conducted in two sessions: one before TMZ treatment and another after the treatment. A 1-minute wait period was observed between the two recording sessions to allow TMZ to diffuse through the sample. Fluorescence fluctuation distributions (DF/F0) were analyzed within a pre-defined region of interest (ROI) using ImageJ 64 software. Data were normalized to the background fluorescence and quantified by calculating the mean of the distribution. Additionally, ΔF/F₀ traces were analysed to identify individual events, and their frequency and duration were measured using custom scripts in MATLAB. Events were defined as fluorescence transients with a minimum duration of 0.5 s to exclude noise-related fluctuations.

### Statistics

All data presented result from the analysis of three or more independent experiments. Statistics was conducted using GraphPad Prism 9. Distribution of the data was determined by the Shapiro-Wilk test and thus quantitative variables were analyzed using either parametric or non-parametric methods. For comparisons between two groups, the t-test was used for normally distributed data, while the Wilcoxon test was applied for non-normally distributed data. Statistical significance is indicated as follows: *p ≤ 0.05, ** *p* ≤ 0.01, *** *p* ≤ 0.001, or **** *p* ≤ 0.0001. The specific test used for each analysis is described in the figure legend.

## Electronic supplementary material

Below is the link to the electronic supplementary material.

Supplementary Material 1

Supplementary Material 2

Supplementary Material 3

Supplementary Material 4

Supplementary Material 5

Supplementary Material 6

## Data Availability

The data that support the findings of this study are available from the corresponding author, upon reasonable request.

## References

[1] Monti, L. D. et al. Metabolic and endothelial effects of Trimetazidine on forearm skeletal muscle in patients with type 2 diabetes and ischemic cardiomyopathy. *Am. J. Physiol. Endocrinol. Metab.* **290**, E54–E59. 10.1152/ajpendo.00083.2005 (2006). 16174656 10.1152/ajpendo.00083.2005

[2] Ferraro, E. et al. Improvement of skeletal muscle performance in ageing by the metabolic modulator Trimetazidine. *J. Cachexia Sarcopenia Muscle*. **7**, 449–457. 10.1002/jcsm.12097 (2016). 27239426 10.1002/jcsm.12097 PMC4864287

[3] Molinari, F. et al. The mitochondrial metabolic reprogramming agent Trimetazidine as an ‘exercise mimetic’ in cachectic C26-bearing mice. *J. Cachexia Sarcopenia Muscle*. **8**, 954–973. 10.1002/jcsm.12226 (2017). 29130633 10.1002/jcsm.12226 PMC5700442

[4] Belli, R. et al. *Metabolic Reprogramming Promotes Myogenesis Dur. Aging Front. Physiol.* **10**, 897. 10.3389/fphys.2019.00897. (2019). 10.3389/fphys.2019.00897 PMC6636331 31354530

[5] Vitale, C. et al. Trimetazidine improves exercise performance in patients with peripheral arterial disease. *Pharmacol. Res.* **63**, 278–283. 10.1016/j.phrs.2011.01.003 (2011). 21220024 10.1016/j.phrs.2011.01.003

[6] Scaricamazza, S. et al. Repurposing of Trimetazidine for amyotrophic lateral sclerosis: A study in SOD1. *Br. J. Pharmacol.* **179**, 1732–1752. 10.1111/bph.15738 (2022). 34783031 10.1111/bph.15738 PMC9305494

[7] Van den Bergh, R., Swerts, L., Hendrikx, A., Boni, L. & Meulepas, E. Adipose tissue cellularity in patients with amyotrophic lateral sclerosis. *Clin. Neurol. Neurosurg.* **80**, 226–239. 10.1016/s0303-8467(78)80013-4 (1977). 216513 10.1016/s0303-8467(78)80013-4

[8] Fayemendy, P. et al. Hypermetabolism is a reality in amyotrophic lateral sclerosis compared to healthy subjects. *J. Neurol. Sci.* **420**, 117257. 10.1016/j.jns.2020.117257 (2021). 33290920 10.1016/j.jns.2020.117257

[9] Ferri, A. & Coccurello, R. What is hyper in the ALS hypermetabolism? *Mediat. Inflamm.**2017*, **7821672**. 10.1155/2017/7821672 (2017). 10.1155/2017/7821672 PMC5610793 29081604

[10] Fragasso, G. et al. Short- and long-term beneficial effects of Trimetazidine in patients with diabetes and ischemic cardiomyopathy. *Am. Heart J.* **146**, E18. 10.1016/S0002-8703(03)00415-0 (2003). 14597947 10.1016/S0002-8703(03)00415-0

[11] Kantor, P. F., Lucien, A., Kozak, R. & Lopaschuk, G. D. The antianginal drug Trimetazidine shifts cardiac energy metabolism from fatty acid oxidation to glucose oxidation by inhibiting mitochondrial long-chain 3-ketoacyl coenzyme A thiolase. *Circ. Res.* **86**, 580–588. 10.1161/01.res.86.5.580 (2000). 10720420 10.1161/01.res.86.5.580

[12] Kalucka, J. et al. Quiescent endothelial cells upregulate fatty acid β-Oxidation for vasculoprotection via redox homeostasis. *Cell. Metab.* **28**, 881–894e813. 10.1016/j.cmet.2018.07.016 (2018). 30146488 10.1016/j.cmet.2018.07.016

[13] Guarnieri, C., Finelli, C., Zini, M. & Muscari, C. Effects of Trimetazidine on the calcium transport and oxidative phosphorylation of isolated rat heart mitochondria. *Basic. Res. Cardiol.* **92**, 90–95. 10.1007/BF00805569 (1997). 9166988 10.1007/BF00805569

[14] Stary, C. M., Kohin, S., Samaja, M., Howlett, R. A. & Hogan, M. C. Trimetazidine reduces basal cytosolic Ca2 + concentration during hypoxia in single Xenopus skeletal myocytes. *Exp. Physiol.* **88**, 415–421. 10.1113/eph8802498 (2003). 12719766 10.1113/eph8802498

[15] Wei, J., Xu, H., Shi, L., Tong, J. & Zhang, J. Trimetazidine protects cardiomyocytes against hypoxia-induced injury through ameliorates calcium homeostasis. *Chem. Biol. Interact.* **236**, 47–56. 10.1016/j.cbi.2015.04.022 (2015). 25937560 10.1016/j.cbi.2015.04.022

[16] Xiao, Z. et al. Trimetazidine affects mitochondrial calcium uniporter expression to restore ischemic heart function via reactive oxygen Species/NFκB pathway Inhibition. *J. Cardiovasc. Pharmacol.* **82**, 104–116. 10.1097/FJC.0000000000001434 (2023). 37163369 10.1097/FJC.0000000000001434 PMC10402877

[17] Oliveira, N. A. S., Pinho, B. R. & Oliveira, J. M. A. Swimming against ALS: how to model disease in zebrafish for pathophysiological and behavioral studies. *Neurosci. Biobehav Rev.* **148**, 105138. 10.1016/j.neubiorev.2023.105138 (2023). 36933816 10.1016/j.neubiorev.2023.105138

[18] Kubat, G. B. & Picone, P. Skeletal muscle dysfunction in amyotrophic lateral sclerosis: a mitochondrial perspective and therapeutic approaches. *Neurol. Sci.* **45**, 4121–4131. 10.1007/s10072-024-07508-6 (2024). 38676818 10.1007/s10072-024-07508-6 PMC11306305

[19] Le Gall, L. et al. Molecular and cellular mechanisms affected in ALS. *J. Pers. Med.* **10**10.3390/jpm10030101 (2020). 10.3390/jpm10030101 PMC7564998 32854276

[20] Turner, M. R. et al. Mechanisms, models and biomarkers in amyotrophic lateral sclerosis. *Amyotroph. Lateral Scler. Frontotemporal Degener*. **14** (Suppl 1), 19–32. 10.3109/21678421.2013.778554 (2013). 23678877 10.3109/21678421.2013.778554 PMC4284067

[21] Rowland, L. P. & Shneider, N. A. Amyotrophic lateral sclerosis. *N Engl. J. Med.* **344**, 1688–1700. 10.1056/NEJM200105313442207 (2001). 11386269 10.1056/NEJM200105313442207

[22] Brunet, A., Stuart-Lopez, G., Burg, T., Scekic-Zahirovic, J. & Rouaux, C. Cortical circuit dysfunction as a potential driver of amyotrophic lateral sclerosis. *Front. Neurosci.* **14**, 363. 10.3389/fnins.2020.00363 (2020). 32410944 10.3389/fnins.2020.00363 PMC7201269

[23] Loeffler, J. P., Picchiarelli, G., Dupuis, L. & Gonzalez De Aguilar, J. L. The role of skeletal muscle in amyotrophic lateral sclerosis. *Brain Pathol.* **26**, 227–236. 10.1111/bpa.12350 (2016). 26780251 10.1111/bpa.12350 PMC8029271

[24] Leal, S. S. & Gomes, C. M. Calcium dysregulation links ALS defective proteins and motor neuron selective vulnerability. *Front. Cell. Neurosci.* **9**, 225. 10.3389/fncel.2015.00225 (2015). 26136661 10.3389/fncel.2015.00225 PMC4468822

[25] De Marco, G. et al. Effects of intracellular calcium accumulation on proteins encoded by the major genes underlying amyotrophic lateral sclerosis. *Sci. Rep.* **12**, 395. 10.1038/s41598-021-04267-8 (2022). 35013445 10.1038/s41598-021-04267-8 PMC8748718

[26] Wainger, B. J. et al. Intrinsic membrane hyperexcitability of amyotrophic lateral sclerosis patient-derived motor neurons. *Cell. Rep.* **7**, 1–11. 10.1016/j.celrep.2014.03.019 (2014). 24703839 10.1016/j.celrep.2014.03.019 PMC4023477

[27] Vucic, S., Rothstein, J. D. & Kiernan, M. C. Advances in treating amyotrophic lateral sclerosis: insights from pathophysiological studies. *Trends Neurosci.* **37**, 433–442. 10.1016/j.tins.2014.05.006 (2014). 24927875 10.1016/j.tins.2014.05.006

[28] Park, S. B., Kiernan, M. C. & Vucic, S. Axonal Excitability in Amyotrophic Lateral Sclerosis: Axonal Excitability in ALS. Neurotherapeutics *14*, 78–90. (2017). 10.1007/s13311-016-0492-9 10.1007/s13311-016-0492-9 PMC5233634 27878516

[29] King, A. E., Woodhouse, A., Kirkcaldie, M. T. & Vickers, J. C. Excitotoxicity in ALS: overstimulation, or overreaction? *Exp. Neurol.* **275 Pt 1**, 162–171. 10.1016/j.expneurol.2015.09.019 (2016). 26584004 10.1016/j.expneurol.2015.09.019

[30] de Carvalho, M., Kiernan, M. C. & Swash, M. Fasciculation in amyotrophic lateral sclerosis: origin and pathophysiological relevance. *J. Neurol. Neurosurg. Psychiatry*. **88**, 773–779. 10.1136/jnnp-2017-315574 (2017). 28490504 10.1136/jnnp-2017-315574

[31] Harley, P. et al. Aberrant axon initial segment plasticity and intrinsic excitability of ALS HiPSC motor neurons. *Cell. Rep.* **42**, 113509. 10.1016/j.celrep.2023.113509 (2023). 38019651 10.1016/j.celrep.2023.113509 PMC7618452

[32] Benedetti, L. et al. INaP selective Inhibition reverts precocious inter- and motorneurons hyperexcitability in the Sod1-G93R zebrafish ALS model. *Sci. Rep.* **6**, 24515. 10.1038/srep24515 (2016). 27079797 10.1038/srep24515 PMC4832213

[33] Devlin, A. C. et al. Human iPSC-derived motoneurons harbouring TARDBP or C9ORF72 ALS mutations are dysfunctional despite maintaining viability. *Nat. Commun.* **6**, 5999. 10.1038/ncomms6999 (2015). 25580746 10.1038/ncomms6999 PMC4338554

[34] Fischer, L. R. et al. Amyotrophic lateral sclerosis is a distal axonopathy: evidence in mice and man. *Exp. Neurol.* **185**, 232–240. 10.1016/j.expneurol.2003.10.004 (2004). 14736504 10.1016/j.expneurol.2003.10.004

[35] Delestrée, N. et al. Adult spinal motoneurones are not hyperexcitable in a mouse model of inherited amyotrophic lateral sclerosis. *J. Physiol.* **592**, 1687–1703. 10.1113/jphysiol.2013.265843 (2014). 24445319 10.1113/jphysiol.2013.265843 PMC3979619

[36] Filipchuk, A. et al. Early hypoexcitability in a subgroup of spinal motoneurons in superoxide dismutase 1 Transgenic mice, a model of amyotrophic lateral sclerosis. *Neuroscience* **463**, 337–353. 10.1016/j.neuroscience.2021.01.039 (2021). 33556455 10.1016/j.neuroscience.2021.01.039

[37] Martínez-Silva, M. L. et al. Hypoexcitability precedes denervation in the large fast-contracting motor units in two unrelated mouse models of ALS. *Elife* **7**. 10.7554/eLife.30955 (2018). 10.7554/eLife.30955 PMC5922970 29580378

[38] Pambo-Pambo, A., Durand, J. & Gueritaud, J. P. Early excitability changes in lumbar motoneurons of Transgenic SOD1G85R and SOD1G(93A-Low) mice. *J. Neurophysiol.* **102**, 3627–3642. 10.1152/jn.00482.2009 (2009). 19828728 10.1152/jn.00482.2009

[39] Quinlan, K. A., Schuster, J. E., Fu, R., Siddique, T. & Heckman, C. J. Altered postnatal maturation of electrical properties in spinal motoneurons in a mouse model of amyotrophic lateral sclerosis. *J. Physiol.* **589**, 2245–2260. 10.1113/jphysiol.2010.200659 (2011). 21486770 10.1113/jphysiol.2010.200659 PMC3098701

[40] Leroy, F., d’Incamps, L., Imhoff-Manuel, B. & Zytnicki, D. R.D. Early intrinsic hyperexcitability does not contribute to motoneuron degeneration in amyotrophic lateral sclerosis. *Elife**3*. (2014). 10.7554/eLife.04046. 10.7554/eLife.04046 PMC4227046 25313866

[41] Vucic, S., Ziemann, U., Eisen, A., Hallett, M. & Kiernan, M. C. Transcranial magnetic stimulation and amyotrophic lateral sclerosis: pathophysiological insights. *J. Neurol. Neurosurg. Psychiatry*. **84**, 1161–1170. 10.1136/jnnp-2012-304019 (2013). 23264687 10.1136/jnnp-2012-304019 PMC3786661

[42] Vucic, S., Pavey, N., Haidar, M., Turner, B. J. & Kiernan, M. C. Cortical hyperexcitability: diagnostic and pathogenic biomarker of ALS. *Neurosci. Lett.* **759**, 136039. 10.1016/j.neulet.2021.136039 (2021). 34118310 10.1016/j.neulet.2021.136039

[43] Saba, L. et al. Modified age-dependent expression of NaV1.6 in an ALS model correlates with motor cortex excitability alterations. *Neurobiol. Dis.* **130**, 104532. 10.1016/j.nbd.2019.104532 (2019). 31302244 10.1016/j.nbd.2019.104532

[44] Kuo, J. J., Siddique, T., Fu, R. & Heckman, C. J. Increased persistent Na(+) current and its effect on excitability in motoneurones cultured from mutant SOD1 mice. *J. Physiol.* **563**, 843–854. 10.1113/jphysiol.2004.074138 (2005). 15649979 10.1113/jphysiol.2004.074138 PMC1665614

[45] Martin, E., Cazenave, W., Cattaert, D. & Branchereau, P. Embryonic alteration of motoneuronal morphology induces hyperexcitability in the mouse model of amyotrophic lateral sclerosis. *Neurobiol. Dis.* **54**, 116–126. 10.1016/j.nbd.2013.02.011 (2013). 23466698 10.1016/j.nbd.2013.02.011

[46] Sareen, D. et al. Targeting RNA foci in iPSC-derived motor neurons from ALS patients with a C9ORF72 repeat expansion. *Sci. Transl Med.* **5**, 208ra149. 10.1126/scitranslmed.3007529 (2013). 24154603 10.1126/scitranslmed.3007529 PMC4090945

[47] Renton, A. E., Chiò, A. & Traynor, B. J. State of play in amyotrophic lateral sclerosis genetics. *Nat. Neurosci.* **17**, 17–23. 10.1038/nn.3584 (2014). 24369373 10.1038/nn.3584 PMC4544832

[48] Naujock, M. et al. 4-Aminopyridine induced activity rescues hypoexcitable motor neurons from amyotrophic lateral sclerosis Patient-Derived induced pluripotent stem cells. *Stem Cells*. **34**, 1563–1575. 10.1002/stem.2354 (2016). 26946488 10.1002/stem.2354

[49] Sances, S. et al. Modeling ALS with motor neurons derived from human induced pluripotent stem cells. *Nat. Neurosci.* **19**, 542–553. 10.1038/nn.4273 (2016). 27021939 10.1038/nn.4273 PMC5015775

[50] Liu, Y. et al. C9orf72 BAC mouse model with motor deficits and neurodegenerative features of ALS/FTD. *Neuron* **90**, 521–534. 10.1016/j.neuron.2016.04.005 (2016). 27112499 10.1016/j.neuron.2016.04.005

[51] Marchand-Pauvert, V. et al. Absence of hyperexcitability of spinal motoneurons in patients with amyotrophic lateral sclerosis. *J. Physiol.* **597**, 5445–5467. 10.1113/JP278117 (2019). 31523813 10.1113/JP278117

[52] Tremblay, E., Martineau, É. & Robitaille, R. Opposite synaptic alterations at the neuromuscular junction in an ALS mouse model: when motor units matter. *J. Neurosci.* **37**, 8901–8918. 10.1523/JNEUROSCI.3090-16.2017 (2017). 28821658 10.1523/JNEUROSCI.3090-16.2017 PMC6596800

[53] Saxena, S. et al. Neuroprotection through excitability and mTOR required in ALS motoneurons to delay disease and extend survival. *Neuron* **80**, 80–96. 10.1016/j.neuron.2013.07.027 (2013). 24094105 10.1016/j.neuron.2013.07.027

[54] Hale, M. E., Katz, H. R., Peek, M. Y. & Fremont, R. T. Neural circuits that drive startle behavior, with a focus on the Mauthner cells and spiral fiber neurons of fishes. *J. Neurogenet.* **30**, 89–100. 10.1080/01677063.2016.1182526 (2016). 27302612 10.1080/01677063.2016.1182526

[55] Jontes, J. D., Buchanan, J. & Smith, S. J. Growth cone and dendrite dynamics in zebrafish embryos: early events in synaptogenesis imaged in vivo. *Nat. Neurosci.* **3**, 231–237. 10.1038/72936 (2000). 10700254 10.1038/72936

[56] Rupprecht, P., Prendergast, A., Wyart, C. & Friedrich, R. W. Remote z-scanning with a macroscopic voice coil motor for fast 3D multiphoton laser scanning microscopy. *Biomed. Opt. Express*. **7**, 1656–1671. 10.1364/BOE.7.001656 (2016). 27231612 10.1364/BOE.7.001656 PMC4871072

[57] Della Vecchia, S. et al. Trehalose Treatment in Zebrafish Model of Lafora Disease. *Int. J. Mol. Sci.**23*. 10.3390/ijms23126874 (2022). 10.3390/ijms23126874 PMC9224929 35743315

