# Supplementary figures and images for: Trimetazidine stimulates intracellular Ca2+ transients and zebrafish locomotor activity in spinal neurons
Source: Sci Rep. 2025 Jul 2;15:22854. doi: 10.1038/s41598-025-06065-y (PMC12214544; doi:10.1038/s41598-025-06065-y)

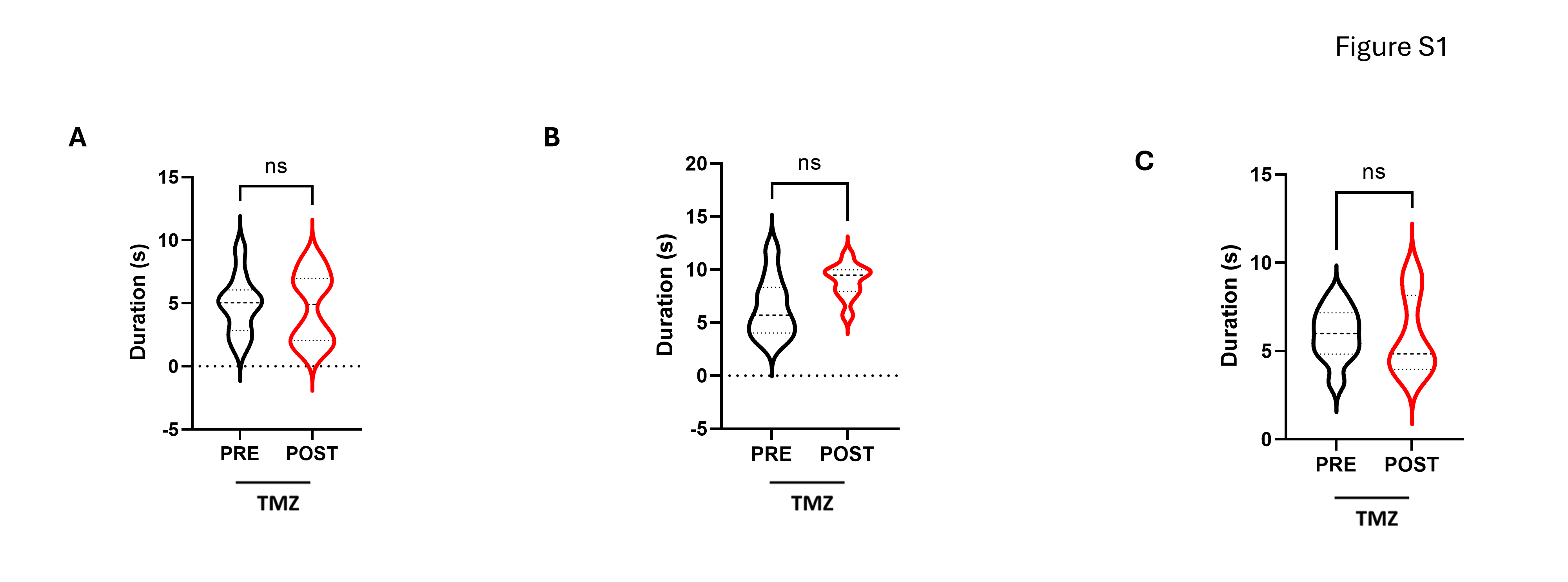

Supplement: Supplementary file 3 — Supplementary Material 3 [file 41598_2025_6065_MOESM3_ESM.tif]

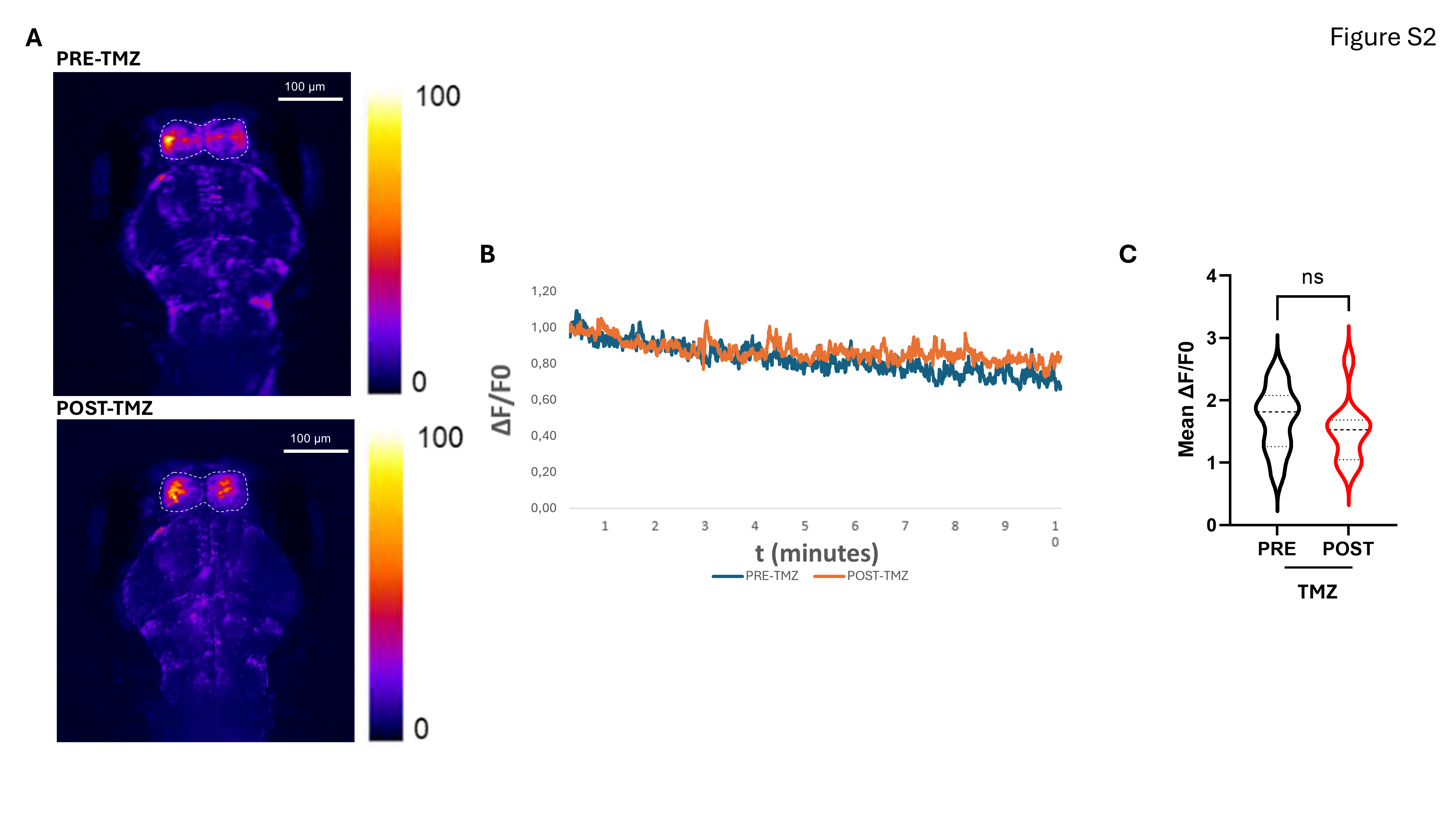

Supplement: Supplementary file 4 — Supplementary Material 4 [file 41598_2025_6065_MOESM4_ESM.tif]
