# Supplementary material for: Trimetazidine stimulates intracellular Ca2+ transients and zebrafish locomotor activity in spinal neurons
Source: Sci Rep. 2025 Jul 2;15:22854. doi: 10.1038/s41598-025-06065-y (PMC12214544; doi:10.1038/s41598-025-06065-y)
Supplement: Supplementary file 6 — Supplementary Material 6 [file 41598_2025_6065_MOESM6_ESM.docx]

**SUPPLEMENTARY MATERIAL CAPTIONS**

**Supplementary video S1. Spinal cord Ca^2+^** **imaging**

Representative recordings of spinal cord neurons Ca^2+^ imaging performed in the lateral caudal neurons of a single zebrafish larva before treatment (PRE-TMZ) and after treatment (POST-TMZ) (100 µM), recordings were speed up at 70 fps with ImageJ.

**Supplementary video S2. Whole brain Ca^2+^ imaging**

Representative recordings of whole brain Ca^2+^ imaging in the rostral-dorsally placed larvae before the treatment (PRE-TMZ) and after the treatment (POST-TMZ) (100 µM), recordings were speed up at 70 fps with ImageJ.

**Supplementary video S3. Hindbrain Ca^2+^ imaging**

Representative recordings of hindbrain Ca^2+^ imaging performed in the larvae rostral-dorsally settled before the treatment (PRE-TMZ) and after the treatment (POST-TMZ) (100 µM), recordings were speed up at 70 fps with ImageJ.

**Supplementary figure S1. Frequency and duration of Ca^2+^ imaging**

**(A)** The graph shows full distribution of data, with median and quartiles of duration of fluorescent signal in spinal neurons**.** n = 10 analyzed larvae. Statistical analysis was performed using the paired Wilcoxon test. ns: not significant (p > 0.9). **(B)** The graph shows full distribution of data, with median and quartiles of duration of fluorescent signal in whole brain**.** n = 10 analyzed larvae. Statistical analysis was performed using the paired Wilcoxon test. ns: not significant (p = 0.08). **(C)** The graph shows full distribution of data, with median and quartiles of duration of fluorescent signal in hindbrain**.** n = 10 analyzed larvae. Statistical analysis was performed using the paired Wilcoxon test. ns: not significant (p = 0.76).

**Supplementary figure S2. Olfactory bulb** Ca^2+^ **imaging upon TMZ exposure**

**(A)** Representative z-projection images of the whole brain and olfactory blub Ca^2+^ imaging performed in the rostral-dorsally placed Tg(*neurod1*:GCaMP6f) larvae before the treatment (PRE-TMZ) and after the treatment (POST-TMZ). On the right side, the look up table color range of Ca^2+^ fluorescence is shown. Images were taken with 4X magnification objective. The dotted line indicates the analyzed ROI. **(B)** A representative graph from a single zebrafish larva shows the ∆F/F0 Ca^2+^ fluorescent signal in the olfactory bulb for each frame before and after TMZ treatment **(C**) The graph shows full distribution of data, with median and quartiles of ∆F/F0 fluorescent signal in olfactory bulb. n = 10 analyzed larvae. Statistical analysis was performed using the paired Wilcoxon test. ns: not significant (p-value = 0.23).
